# Assessment of physicians’ awareness and knowledge of familial hypercholesterolemia in Saudi Arabia: Is there a gap?

**DOI:** 10.1371/journal.pone.0183494

**Published:** 2017-08-17

**Authors:** Mohammed Ali Batais, Turky H. Almigbal, Aref A. Bin Abdulhak, Hani B. Altaradi, Khalid F. AlHabib

**Affiliations:** 1 Department of Family and Community Medicine, King Saud University, Riyadh, Saudi Arabia; 2 Division of Cardiovascular Disease, Department of Internal Medicine, University of Iowa Hospitals and Clinics, Iowa City, Iowa, United States of America; 3 Department of Cardiac Sciences, King Fahad Cardiac Center, College of Medicine, King Saud University, Riyadh, Saudi Arabia; TNO, NETHERLANDS

## Abstract

**Background:**

The scarcity of familial hypercholesterolemia (FH) cases reported in Saudi Arabia might be indicative of a lack of awareness of this common genetic disease among physicians.

**Objective:**

To assess physicians’ awareness, practice, and knowledge of FH in Saudi Arabia.

**Methods:**

This is a cross-sectional study conducted among physicians at four tertiary hospitals in Riyadh, Saudi Arabia between March 2016 and May 2016 using a self-administered questionnaire.

**Results:**

A total of 294 physicians completed the survey (response rate 90.1%). Overall, 92.9% of the participants have poor knowledge of FH while only 7.1% have acceptable knowledge. The majority (68.7%) of physicians rated their familiarity with FH as average or above average, and these had higher mean knowledge scores than participants with self-reported below average familiarity (mean 3.4 versus 2.6) (P < 0.001). Consultant physicians were 4.2 times more likely to be familiar with FH than residents or registrars (OR = 4.2, 95% CI = 1.9–9.1, P < 0.001). Physicians who currently managed FH patients had higher mean knowledge scores compared to those without FH patients in their care (3.5 versus 2.9) (P = 0.006). In addition, there were statistically significant differences between physicians’ mean knowledge scores and their ages, levels of training, and years in practice. Moreover, a substantial deficit was identified in the awareness of various clinical algorithms to diagnose patients with FH, cascade screening, specialist lipid services, and the existence of statin alternatives, such as proprotein convertase subtilisin/kexin type 9 (PCSK9) inhibitors.

**Conclusion:**

A substantial deficit was found in the awareness, knowledge, practice, and detection of FH among physicians in Saudi Arabia. Extensive educational programs are required to raise physician awareness and implement best practices; only then can the impact of these interventions on FH management and patient outcome be assessed.

## Introduction

Familial hypercholesterolemia (FH) is a preventable cause of premature coronary artery disease (CAD). FH is a monogenic autosomal dominant inherited disorder of lipid metabolism characterized by elevated low-density lipoprotein cholesterol (LDL-C) levels and a very high risk of premature CAD [1]. Untreated patients with FH have a 20-fold increased risk of cardiovascular events, a leading cause of mortality in the Middle East [1, 2].

Several tools are available to diagnose adult patients with FH, such as the Make Early Diagnosis to Prevent Early Death [3], Simon Broome [4], and Dutch Lipid Clinic Network (DLCN) criteria [5]. Although these can all help to detect FH cases, the DLCN criteria have been widely used due to their higher sensitivity [6, 7]. Damgaard et al. [8] tested the ability of these three clinical criteria to predict the results of molecular genetic analysis. Mutation detection rates (and specificities) are high only if sensitivity is very low and vice versa. The detection rates in patients receiving a definite FH clinical diagnosis by the Simon Broome criteria were 61.3% and 62.9% by the Dutch Lipid Clinic Network criteria. However, in patients receiving a clinical diagnosis of possible FH based on the molecular genetic analysis, the Simon Broome criteria resulted in a high sensitivity of 90.4% and Dutch Lipid Clinic Network criteria of 99.3% with correspondingly lower mutation detection rates of 38.5% and 34.3%, respectively. Genetic testing may also be used in the diagnosis of FH and to determine the pattern of inheritance (heterozygous or homozygous) [9]. Cascade screening involves lipid and/or genetic testing of the relatives of an index case diagnosed with FH [10].

Recent FH prevalence estimates have been documented in the United States (1 in 250) [11], China (1 in 213) [12], and Australia (1 in 353-229) [13], however, the magnitude of the problem is largely unknown in the Middle Eastern region, including Saudi Arabia [14]. Three Western countries reported over 500 mutations, whereas only 57 mutations were reported from 17 Middle Eastern and North African (MENA) countries [15]. Opportunistic screening by general practitioners (GPs) could address the low reporting of FH and subsequently improve the patient outcome [7, 16–18]. Around 92% of the lipid profiles in the community were requested by GPs, confirming that they play an essential role in detecting individuals with FH [19]; however, several studies have found physicians' knowledge and awareness of FH to be suboptimal [20–23]. The knowledge and awareness of FH between varying specialties (primary care physicians (PCP) versus specialists) was not significantly different, except that about two- thirds of PCPs selected themselves as the best health providers for detecting FH and subsequent screening of the first-degree relatives [20].

The International FH Foundation has recently offered guidance on FH management, from an international perspective, by Watts et al. [24]. This systemic and strategic guidance was recently proposed as an appropriate guide for the establishment of the MENA FH registry [15]. Implementation of such guidance requires an assessment of the current knowledge and practices regarding FH in the region. In this study, we sought to assess several aspects of the awareness, practice, and knowledge of FH among physicians in Saudi Arabia, and compare FH familiarity with physician characteristics, including age, gender, medical specialty, years of practice, and level of training.

## Materials and methods

### Study design and setting

This is a cross-sectional study conducted among physicians using a self-administered questionnaire. The study was conducted at four tertiary hospitals in Riyadh, Saudi Arabia. The study was approved by the Institutional Review Board, College of Medicine, King Saudi University (no. E-16-1824). Each participant that received the questionnaire was informed about the objective of the present study, the Institutional Review Board has approved that filing the survey is sufficient to imply consent.

### Participants and survey instrument

We approached physicians in various clinical specialties including family medicine, internal medicine, cardiology, endocrinology, pediatrics, and obstetrics and gynecology in Riyadh, Saudi Arabia between March 2016 and May 2016. Certified family physicians in Saudi Arabia complete four years of residency training in which they are exposed to 13 specialties in addition to a family medicine practice. They provide care for a wide range of health issues throughout a patient's life cycle in a variety of healthcare settings. The clinical responsibilities of family physicians include general health promotion, disease prevention, screening and diagnosis, and acute and chronic disease management.

All physicians present at the time of data collection in four tertiary hospital sites were included; a paper copy of the questionnaire was delivered to the available physicians during their routine weekly educational activities. Surveys were administered by three medical students trained in data collection, who have received a letter of appreciation as an acknowledgment for their contribution to this study. Before seeing the survey, the physicians were asked to complete an anonymous self-administered survey in English in order to assess their basic background knowledge.

We used a questionnaire originally designed by Bell et al. [21] and Pang et al. [20]. Additional questions were added based on previous expert recommendations and international guidelines dealing with FH management [7, 16, 24], which include awareness of clinical diagnostic algorithms to diagnose patients with FH, awareness of new statin-free treatment options, and LDL-C targets for FH cases with or without known CAD or diabetes.

A panel of two family physicians and one cardiologist assessed the questionnaire for appropriateness, accuracy, and relevance and were asked to criticize the questionnaire’s content. All members of this panel had provided clinical care for FH patients and were familiar with FH guidelines and the survey's development. The questionnaire was initially tested by 20 physicians at King Khalid University Hospital in Riyadh, Saudi Arabia to ensure the questions were clear, understandable, and in logical order. A month later the questionnaire was re-administered to the same group to ensure reliability and consistency in participant response. We used the Kappa test agreement measures between test and retest, and the average kappa value was 0.85 (P <0.001). The reliability coefficient (Cronbach’s alpha) of the questionnaire obtained in this pilot study was >0.7. The 20 baseline questionnaires from this pilot study were excluded from the main study and not further analysed.

The self-administrated questionnaire recorded physician’s demographic data and inquired about the following aspects of FH: familiarity with the condition; the clinical description, prevalence, inheritance, and cardiovascular disease risk factors (such as diabetes, smoking, and elevated plasma lipoprotein(a) (Lp(a)); definitions of premature cardiovascular disease (CVD); clinical features of FH; LDL-C targets for adults with FH, with or without known CAD or diabetes; awareness of the clinical diagnostic algorithms to diagnose patients with FH and cascade screening for patients with FH; whether the diagnosis requires genetic confirmation; methods for alerting the possibility of FH; which health professional is best placed to detect FH; whether they diagnosed, followed, and performed family screening for patients with FH; their knowledge regarding treating patients with severely elevated cholesterol; and their awareness of the new medications for FH. The survey consisted mostly of multiple-choice questions but also included a few open-ended and Yes/No questions (S1 Appendix).

### Sample size

The sample size was calculated based on the results of the pilot study, where 77% of participants reported their familiarity with FH as average and above average. The appropriate sample size was calculated to be 272 participants based on a 5% margin of error, a confidence interval of 95%, and 77% average or above average familiarity with FH. Taking into account the non-response rate of 20%, the survey questionnaire was distributed to 326 participants.

### Statistical tests

Data from the returned questionnaires were coded and entered into the Statistical Package for the Social Sciences (SPSS) software (SPSS Inc., Chicago, IL, USA), which was used for statistical analysis [25]. Descriptive statistics, including frequency distribution and percentages, were applied to both the demographic data and responses to each question. Familiarity with FH among physicians was assessed by a 7-point scale in which 1 meant “not at all familiar” and 7 meant “extremely familiar.” The responses were then classified into “below average” and “average and above” familiarity, where average and above average familiarity was defined for responses of 4–7. The chi-square test was then used to assess the differences between FH familiarity and physicians’ demographics. For a logistic regression analysis, the participants’ familiarity of FH was categorized as a binary outcome, comparing those who are “below average” with those who reported “average and above” familiarity with FH. For the purpose of analysis, participant ages were categorized into three groups: 30 years and less, 31–45 years, or more than 45 years. Similarly, years of practice were categorized as 5 years or less, 6–10 years, 11–15 years, or more than 15 years. Eleven questions assessed physicians’ knowledge of FH. For each question, a correct answer was scored as 1 point, while an incorrect answer was given a score of 0. A mean knowledge score was computed by summing the correct answers to all 11 questions; possible total mean scores ranged from 0 to 11. The knowledge regarding FH was considered acceptable if the total score was >50%. Testing the normality distribution of the knowledge score revealed a normal distribution; hence, Student’s t- and ANOVA tests were used to compare the mean knowledge score with a physician’s specialty, demographics, and their familiarity with FH. Significance was defined at the two-tail P value of 5% for all analyses.

## Results

### Sample characteristics

A total of 326 surveys were distributed to the physicians, and 294 completed the survey (response rate 90.1%). Of the 294 physicians, 175 (59.5%) were male. The participants' age range was (27–63) years, the mean age was 36.5 years (SD ± 9.3), and the mean years of clinical practice was 10.4 years (SD ± 8.1). The majority of the physicians (68.7%) rated their familiarity with FH as average and above. A summary of the physician demographic characteristics is presented in Table 1.

**Table 1:** Physicians’ demographics (n = 294).

Characteristics	N (%)	Mean (SD)
**Age**		36.5 (9.3)
**Gender**		
Male	175 (59.5)	
Female	119 (40.5)	
**Medical Specialty**		
Family Medicine	111 (37.8)	
Cardiology	35 (11.9)	
Endocrinology	23 (7.8)	
Obs/Gyn	27 (9.2)	
Internal Medicine	69 (23.5)	
Pediatric	29 (9.9)	
**Level of training**		
Resident	115 (39.1)	
Registrar	97 (33)	
Consultant	82 (27.9)	
**Years of Practice**		10.4 (8.1)
**Familiarity with FH** *		
Below average	92 (31.3)	
Average and above	202 (68.7)	
**Overall knowledge of FH** **		3.2 (1.7)

Abbreviation: N: number, SD: standard deviation, FH: familial hypercholesterolemia.

* The responses were classified into “below average” and “average and above” familiarity, where average and above familiarity with FH was defined for responses of 4 and above on the 7-point scale.

** A mean knowledge score was computed by summing correct answers to all 11 knowledge questions.

### Knowledge of FH management

The overall mean knowledge score was 3.2 ± 1.7, as shown in Table 1. The clinical description of FH was underestimated by almost a third (32.3%) of the participants. The prevalence of heterozygous FH in the general population as 1:500 was correctly identified by 22.8% of the physicians, though only a third (33%) recognized its pattern of inheritance. Five percent of physicians identified the CVD risk in untreated FH patients as 20 times greater than that of the general population; 90.8% were unable to identify the age threshold for premature CVD in males and 91.8% could not identify it in females. Only 38.8% selected LDL-C < 2.5 mmol/L (< 100 mg/dL) as the target for adults with FH, while 44.6% selected LDL-C < 1.8 mmol/L (< 70 mg/dL) as the target for FH adults with known CAD or diabetes.

The first-line medication to treat FH patients is statins, which was selected by 206 (70.1%) of the physicians. The preferred combination to treat severe hypercholesterolemia was statins plus ezetimibe, which was selected by 38.1% of physicians (Table 2).

**Table 2:** Summary of physicians’ responses to questions about FH knowledge, practice, detection, and awareness.

	N (%)
**Knowledge**	
Correctly described FH	199 (67.7)
Correctly identified the prevalence of heterozygous FH in the general population	67 (22.8)
Correctly identified the transmission rate to first degree relatives	97 (33)
Correctly identified the CVD risk in untreated FH	15 (5.1)
Correctly identified the age threshold for premature CVD in males	27 (9.2)
Correctly identified the age threshold for premature CVD in females	24 (8.2)
Correctly identified that genetic testing was not required to accurately diagnosis FH	137 (46.6)
Correctly identified LDL-C target for adult with FH	114 (38.8)
Correctly identified LDL-C target for FH adults with known CAD or diabetes	131 (44.6)
Selected statins to treat familial hypercholesterolemia	206 (70.1)
Selected a combination of statin and ezetimibe to treat severe hypercholesterolemia	112 (38.1)
**Practice**	
Routinely take a detailed family history, perform physical examination and screen close relatives for all patients with premature CAD	179 (60.9)
Had diagnosed patient with FH	98 (33.3)
Had managed FH patients under their care	103 (35)
Performed routine close relative screening with lipid profile of patients with FH	153 (52)
The most prevalent age for screening young people in kindred with FH was 13–18 years; which was selected by	122 (41.5)
**Opinions and detection**	
Selected family physicians as the most effective health care provider for the early detection of FH	253 (86.1)
Selected laboratory comment on lipid profiles, alert by the clinical software system, and direct telephone call from the laboratory to highlight patients at risk of FH	125 (42.5)
**Awareness**	
Aware of the clinical algorithms to diagnose patients with FH The Simon Broome criteria The Dutch Lipid Clinic Network DLCN criteria The US MedPed Program	58 (19.7) 46 (15.6) 43 (14.6)
Aware of the cascade screening for patients with FH	76 (25.9)
Aware of any specialist clinical services for lipid disorders to whom patients can be referred	136 (46.3)
Aware of the new medications for FH patients beside statins PCSK9 inhibitors Lomitapide (MTP) inhibitors Mipomersen (an antisense oligonucleotide inhibitors)	68 (23.1) 52 (17.7) 35 (11.9)

Abbreviation: N: number, FH: familial hypercholesterolemia.

### Detecting FH

The majority of participants (86.1%) selected family physicians as the most effective at detecting early FH and screening first-degree relatives, followed by cardiologists (5.8%). Laboratory comments on lipid profile alerting a possible FH, alerts by the clinical software system, and direct telephone call from the laboratory were all selected as the preferred choice (42.5%) to help physicians in detecting FH during practice (Table 2).

### Management practices

Routine patient screening including taking a detailed family history, performing a physical examination, and screening the close relatives of all patients with premature CAD was performed by 179 (60.9%) physicians; however, routine close relative lipid profile screening of patients with FH was only chosen by 153 (52%). The most prevalent age for screening young people for FH was 13–18 years; this was selected by 122 (41.5%) physicians. Eighty-nine (33.3%) physicians have diagnosed patients with FH, and (35%) have managed FH patients during their practice. Only 46.6% of physicians recognized that genetic testing was not required to accurately diagnose FH (Table 2).

**Fig 1:**
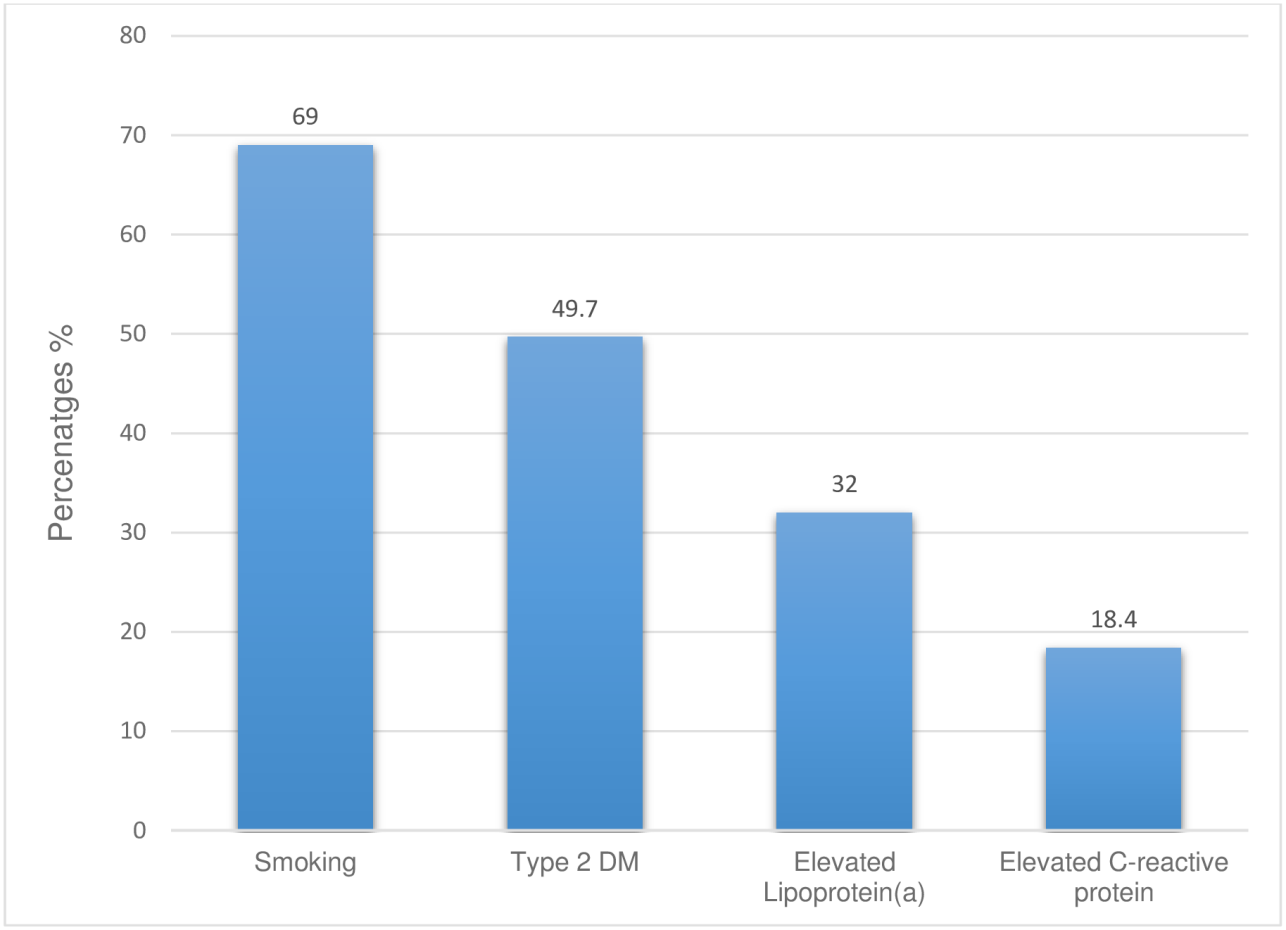
Summary of physician responses to the most selected risk factors that further increase the CV risk of patients with FH.

Overall, 69% of participants identified smoking as a risk factor that further increased CVD risk in FH, 49.7% selected type 2 diabetes, and 32% selected elevated Lp(a) (Fig 1).

### Awareness of FH management

Awareness of various clinical algorithms to diagnose patients with FH was low; 14.6% identified the US MedPed Program, 15.6% the DLCN criteria, and 19.7% the Simon Broome criteria. Only 25.9% of the surveyed physicians were aware of cascade screening for patients with FH, and 46.3% were aware of specialized clinical services for lipid disorders to which patients can be referred. The majority of participants were unaware of the existence of new medications for FH patients besides statins, such as PCSK9 inhibitors, Lomitapide (MTP) inhibitors, and Mipomersen (an antisense oligonucleotide inhibitor) (Table 2).

### Relationship between familiarity and mean knowledge scores of FH and physician demographics

Two hundred and two (68.7%) physicians rated their familiarity with FH as “average and above”. Overall, 92.9% of the participants have poor knowledge regarding FH while only 7.1% have acceptable knowledge. Of the 7.1% participants who have acceptable knowledge, only 8.9% of those reported their FH familiarity as average and above (P = 0.092). A significant association was also found between FH familiarity and the physician’s age, medical specialty, level of training, and years of practice (Table 3).

**Table 3:** The relationship between FH familiarity and physicians’ demographics.

	Familiarity with FH **		
Characteristics	Below average 92 (31.3%) N (%)	Average and above 202(68.7%) N (%)	P value *
**Age**			
≤ 30 years	44 (47.8)	48 (52.2)	<0.001
31–45 years	35 (24.1)	110 (75.9)	
> 45 years	13 (22.8)	44 (77.2)	
**Gender**			
Male	49 (28)	126 (72)	0.140
Female	43 (36.1)	76 (63.9)	
**Medical Specialty**			
Family physicians	21 (18.9)	90 (81.1)	<0.001
Specialized physicians ***	71 (38.8)	112 (61.2)	
**Level of training**			
Resident	53 (46.1)	62 (53.9)	<0.001
Registrar	26 (26.8)	71 (73.2)	
Consultant	13 (15.9)	69 (84.1)	
**Years of Practice**			
≤ 5 years	43 (44.3)	54 (55.7)	0.001
6–10 years	26 (29.5)	62 (70.5)	
11–15 years	5 (10.6)	42 (89.4)	
> 15 years	18 (29)	44 (71)	
**Overall knowledge proportion of FH**^!^			
Acceptable knowledge	3 (3.3)	18 (8.9)	0.092
Poor knowledge	89 (96.7)	184 (91.1)	

* Chi square test was used in the analysis.

** The responses were classified into “below average” and “average and above” familiarity, where average and above familiarity was defined for responses of 4 and above on the 7-point scale.

***Specialized physicians include cardiologists, endocrinologists, gynecologists, internists, and pediatricians.

^!^ The knowledge regarding FH was considered acceptable if the total score was greater than 50%.

Logistic regression analysis, after adjustment of all these variables, revealed that consultant physicians are 4.2 times more likely to be familiar with FH than residents or registrars (OR = 4.2, 95% CI = 1.9–9.1, P < 0.001); however, specialized physicians are 63% less likely to be familiar to FH than family physicians (OR = 0.37, 95% CI = 0.18–0.73, P < 0.004) (Table 4).

**Table 4:** Familiarity with FH as predicted by physicians' characteristics.

	Adjusted		
Characteristics	OR	P-value	95% CI
**Age**			
≤ 30 years (ref)*	-	-	-
31–45 years	1.3	0.60	0.48–3.5
> 45 years	1	0.95	0.23–4.7
**Gender**			
Male (ref)*	-	-	-
Female	0.65	0.13	0.37–1.1
**Medical Specialty**			
Family physicians (ref)*	-	-	-
Specialized physicians	0.37	0.004	0.1–0.73
**Level of training**			
Resident (ref)*	-	-	-
Registrar	1.65	0.127	0.8–3.1
Consultant	4.2	<0.001	1.9–9.1
**Years of Practice**			
≤ 5 years (ref)*	-	-	-
6–10 years	0.66	0.392	0.27–1.6
11–15 years	1.8	0.371	0.49–6.4
> 15 years	0.57	0.292	0.19–1.6

* Ref: reference (for categorical covariate in logistic regression analysis one of the categories is considered as reference category. Then the odds ratio (OR) calculated for each of the other categories with respect to the reference category).

Though the difference was not statistically significant, family physicians have higher knowledge scores than specialized physicians (mean 3.4 versus 3) (P = 0.053). Physicians with average and above familiarity with FH had higher mean knowledge scores than those with below average familiarity (mean 3.4 versus 2.6) (P < 0.001). Physicians who currently managed FH patients had higher mean knowledge scores compared to those who reported no FH patients in their care (3.5 versus 2.9) (P = 0.006). Moreover, there were statistically significant differences between physicians’ mean knowledge scores and their ages, levels of training, and years in practice. The mean knowledge scores were higher among registrars and consultants, in the 31 to 45 year age groups, and with practice duration between 6–10 years or >15 years (Table 5).

**Table 5:** Mean knowledge score by physicians’ characteristics and FH familiarity.

Characteristics	N	Mean Knowledge score	P value*
**Medical Specialty**			
Family Medicine	111	3.43	0.053
Specialized physicians **	183	3.03	
**Familiarity with FH**			
Below average	92	2.6	<0.001
Average and above	202	3.4	
**Gender**			
Male	175	3.1	0.98
Female	119	3.1	
**Manage FH patients**			
Yes	103	3.5	0.006
No	190	2.9	
**Level of training**			
Resident	115	2.6	<0.001
Registrar	97	3.5	
Consultant	82	3.5	
**Years of Practice**			
≤ 5 years	97	2.6	0.001
6–10 years	88	3.5	
11–15 years	47	3.1	
> 15 years	62	3.5	
**Age**			
≤ 30 years	92	2.7	0.007
31–45 years	145	3.4	
> 45 years	57	3.3	

* Student t-test and ANOVA test were used in the analysis.

**Specialized physicians include cardiologists, endocrinologists, gynecologists, internists, and pediatricians.

## Discussion

The overall prevalence of FH in the Middle East is largely unknown due to the lack of registries and cascade genetic screening. The underreporting of FH mutations in the region where the consanguinity rate is high indicates poor awareness of CVD genetic risk [26]. Although most physicians perceived their familiarity with FH as average and above average, important deficits were identified. Two-thirds of surveyed physicians were able to define FH correctly, but knowledge of the prevalence and heritability of FH as well as the definition of premature CVD was very low. Moreover, this study revealed substantial deficits in the awareness of various clinical algorithms to diagnose patients with FH, cascade screening, specialist lipid services, and the existence of new, non-statin medications for FH, such as PCSK9 inhibitors. Our findings are consistent with previous studies [20–23].

International guidelines label FH patients as having high cardiovascular (CV) risk; therefore, the optimal LDL-C goal should be < 2.5 mmol/L (< 100 mg/dL) or < 1.8 mmol/L (< 70 mg/dL) with known atherosclerotic cardiovascular disease (ASCVD), or at least a 50% reduction in LDL-C levels [10, 24]. Over half of the physicians in this study were unaware of the optimal target LDL-C levels of FH patients. Achievement of such targets is imperative towards reducing lifetime CVD risk [27]; however, only a minority of FH patients reach their optimal treatment goals [28]. Even with high doses of the most potent statins, target LDL-C levels are not achieved in many patients [10]. In the Centralized Pan-Middle East Survey (CEPHEUS), which was conducted over six Persian Gulf countries, only 52% of patients attained their LDL-C goals [29]. Failure to reach optimal targets among high and very high CV risk patients with dyslipidemia may be related to both patient and physician factors. There is a documented lack of awareness and poor adherence to general dyslipidemia guidelines by physicians [26]. Physicians also seldom use high doses of statins in high-risk patients, and the majority of patients remained on the same dose of statin initially prescribed [29]. Taken together this data indicates that education should emphasize not only the importance of screening, but also the need for aggressive treatment paradigms [30].

Knowledge of potential therapeutic options for FH is crucial for appropriate treatment. Despite the development of novel therapeutics that are highly effective in lowering LDL-C, illustrated by increased use of PCSK9 inhibitors [31], the majority of our sample was unaware of these new medications, and only 38.1% would add ezetimibe to traditional statin treatment in severe cases of hypercholesterolemia. Our data is consistent with another study that reported only 23.4% of respondents were aware of PCSK9 inhibitors as a potential therapeutic option [22]. The importance of optimizing the treatment of high-risk patients by providing moderate to high doses of statin plus ezetimibe and increasing the familiarity of treating providers to the new therapeutic options for FH patients, such as PCSK9 inhibitors, was recognized previously [26].

Early treatment is mandatory in preventing CVD among FH patients, as they have a 20-fold higher risk of developing premature CAD [2]; however, this risk reduces to that of the general population if FH patients are effectively treated [32]. In our survey, more than 90% of the physicians were unable to identify the CVD risk in untreated FH patients as 20 times greater than the general population, and were unable to identify the age threshold for premature CAD in both sexes. FH should always be considered when a family or personal history of premature CVD is presented, and the inability of physicians to recognize the correct premature age for CVD may result in inadequate detection of FH in both primary and secondary settings [22].

A survey among 191 GPs in Western Australia reported that the majority of GPs described themselves as the most effective healthcare provider to detect FH, which encourages GPs to contribute to both opportunistic and systematic FH detection; however, this survey shows that GPs’ awareness and knowledge of FH were suboptimal [21]. In our study, family physician was selected by 86.1% as the most effective health care provider for detecting early FH and screening first-degree relatives, followed by cardiologists (5.8%), consistent with previous studies conducted in the United Kingdom (UK) [22] and Asia [20]. Primary care physicians are still the best-placed physicians to identify FH in the primary prevention setting, while the role of specialized physicians, such as cardiologists and endocrinologists, are positioned to identify FH in a secondary prevention setting [20]. As the detection and management of well-controlled and low complexity cases of FH can take place at the primary care setting, education and training of family physicians regarding lipid management should be initiated.

Early detection and diagnosis of FH prior to the development of complications is essential for the management of FH. Very low awareness (<20%) among our respondents of various clinical algorithm tools was reported in this survey with only 25.9% aware of cascade screening for patients with FH. This awareness is lower than reported in the UK, where it was found that 43.5% of physicians were aware of the Simon Broome diagnostic criteria and 65.9% were aware that family cascade screening is recommended by National Institutes for Health and Care Excellence (NICE) guidelines [22]. This is important because cascade screening of first-degree relatives of index cases is documented to be the most cost-effective method to identify previously undiagnosed FH subjects [33]. However, just 33% of our physicians recognize the pattern of FH inheritance, lower than recently reported among physicians in Asia (47%) [20], and in the UK (50.5%) [22]. This may partially explain the under-appreciation and under-diagnosis of FH cases and the role of cascade screening. Further work and research is required to raise awareness and develop various clinical tools for FH detection at primary care settings to minimize the direct and indirect financial burden on health care systems. Laboratory comments on lipid profiles to alert a possible FH, alerts by the clinical software system, and direct telephone communication from the laboratory are all choices that would help physicians in detecting FH.

Despite that around a third of the physicians in this study either diagnosed or had managed patients with FH, there was a significant gap in the knowledge and awareness of physicians of FH, which is likely contributing to the under-diagnosis and treatment of FH in Saudi Arabia. Likewise, Pang et al. [20] surveyed 230 primary care and specialist physicians from three developed Asian countries; 47% were aware of the heritability, 27% of the prevalence, and 13% of the risk of cardiovascular disease related to FH. This study recognized significant gaps in FH awareness and knowledge in Asia [20]. One possible reason for our study findings may be related to the lack of developed guidelines for the management of dyslipidemia in the Middle East [26].

An elevated Lp(a) level is a causal risk factor for atherosclerotic cardiovascular disease (ASCVD) [34, 35]. Only one third of Saudi physicians in this survey were able to recognize increased Lp(a) as an adjunctive risk factor in FH patients. This contradicts other surveys, which have reported more awareness of Lp(a) involvement among Korean (83%), Taiwanese (66%), and Japanese physicians (51%) [20]. Establishing a MENA FH registry successfully would positively impact population FH awareness in families and physicians and awareness of CVD risks associated with FH such as diabetes, smoking, and elevated Lp(a) levels [15]. Attention to the premature CVD risk associated with FH will ultimately drive an increase in cascade screening. A strong pre-registry campaign (e.g. in the media) can achieve these objectives, and could identify any cultural barriers hindering the establishment of a registry [36]. Also, the results of this registry can be fed back to physicians in the form of publications, audits, and conferences to engender a more personalized approach to improving patient care.

FH registry can help in determining the genetic predisposition of such condition which would help in providing an early intervention. Also, it will enable identifying the most common disease-causing mutations among the Saudi population and identify population specific novel mutations helping in future rapid molecular diagnosis [36]. The researchers can access de-identified data of FH database through research ethics committees or the consultative body to determine the burden of disease, and health outcomes. In addition, the careful documentation of FH cases can provide statically improved studies, meta-analytical studies, scientific collaborations, and international clinical trials to take place in Saudi Arabia [36]. Further studies on the natural history of the disease within the Saudi population can be conducted based on the results of this registry; subsequently, this would provide strategic evidence for therapies and suggest methods for economic improvement in healthcare. Furthermore, a comprehensive FH database will support and justify universal screening of FH in children (ages 2–9 years) in Saudi Arabia as has been implemented in other countries [36].

Although our specialized physicians are 63% less likely to be familiar with FH than family physicians, we found no significant differences in FH knowledge scores between family and specialized physicians; the latter finding is also consistent with the results of Pang et al. [20]. The fact that on average, only 7.1% of the participants have an acceptable FH knowledge raises serious concerns. This poor knowledge was observed mainly in the areas of prevalence, inheritance, CVD risk, definitions of premature CVD, LDL-C targets for adults with FH, and the need for genetic confirmation. Our findings are also in consistent with the results of Bell et al. [21] who found knowledge deficits among physicians in prevalence, inheritance, clinical FH feature, and the need for genetic confirmation. However, the association between FH knowledge and physicians' characteristics has not been assessed in previous surveys. We found that the FH knowledge scores tended to be higher among physicians with higher levels of training, longer years in practice, and those currently managing FH patients. Taken together, these data suggest that physicians' experience has an effect on improving their ability to effectively manage FH.

Our study findings support the need for FH ongoing educational programs, which should be directed to all physicians involved in the management of FH patients. Education programs implemented in the UK significantly improved physicians’ knowledge in all aspects of FH management, including the importance of cascade screening [22]. As our senior physicians were more likely to be familiar with FH than residents or registrars, one possible suggestion is to include FH topics in the curricula of various residency and training programs in Saudi Arabia. We recommend that such topics should be mandatory in the curriculum of medical school, as the practice patterns obtained during the training periods set the basis for lifelong practice. Also, educational activities about FH should be disseminated to the public. Affected family members and interested public can attend a variety of public campaigns about FH in addition to using social networking sites to increase public awareness about FH.

The study has some limitations; as a survey, these are mostly limitations in scope, where results show associations and not necessarily causal relationships. Also, physicians were from four tertiary care settings in the capital city of Saudi Arabia, and therefore the results may not be generalizable. However, no similar work has been done in the region and therefore these findings are of general interest and of value going forward.

## Conclusion

A substantial deficit was found in the awareness, knowledge, practice, and detection of FH among physicians in Saudi Arabia. Physicians with average and above average familiarity with FH had higher mean knowledge scores; therefore, further effort is needed to enact extensive educational programs for physicians.

## Supporting information

S1 Appendix Survey questionnaire. (PDF)
