# Supplementary material for: Assessment of physicians’ awareness and knowledge of familial hypercholesterolemia in Saudi Arabia: Is there a gap?
Source: PLoS One. 2017 Aug 17;12(8):e0183494. doi: 10.1371/journal.pone.0183494 (PMC5560711; doi:10.1371/journal.pone.0183494)
Supplement: S1 Appendix — (PDF) [file pone.0183494.s001.pdf]

Questionnaire on knowledge and awareness of familial hypercholesterolemia (FH) administered to Saudi physicians.

The survey is anonymous and participation is not obligatory.

<u>Section 1: Physician's Demographics</u>

1. **What is your age?**

   ____ Years

2. **What is your gender?**

- o Male
- o Female

3. **What is your primary medical specialty?**

- o Family Medicine
- o Cardiology
- o Endocrinology
- o OB/GYN
- o Internal medicine
- o Pediatrics

4. **What is your level of training?**

- o Resident
- o Registrar
- o Consultant

5. **How many years have you been in practice since completing your medical school?**

____ # of years

<u>Section 2:</u>

6. **How familiar are you with familial hypercholesterolemia? Please use a 10-point scale where 1 means "*Not at all familiar*" and 10 means "*Extremely familiar*".**

1<br>Not at all familiar	2	3	4	5	6	7<br>Extremely familiar

The following questions are specific to familial hypercholesterolemia. Please respond to the best of your knowledge, and if you are unsure on any question, select "Don't know."

7. **Which <u>one</u> description below <u>best</u> describes familial hypercholesterolemia?**

- o The presence of family members with diagnosed high cholesterol
- o A genetic disorder that is characterized by very high cholesterol and a family history of premature heart disease
- o The presence of multiple lipid abnormalities that may be genetic in nature
- o An ultra-rare, potentially fatal condition caused by cholesterol levels that can be up to six times the normal level
- o Don't know

8. **What is the prevalence of heterozygous familial hypercholesterolemia in general population?**

- o 1 in 100 persons
- o 1 in 500 persons
- o 1 in 1,000 persons
- o 1 in 2,000 persons
- o 1 in 5,000 persons
- o Don't know

9. **What is the likelihood that first-degree relatives (i.e. parents, siblings and children) of someone who has familial hypercholesterolemia will also have FH themselves?**

- o 0%
- o 25%

- 50%
- 75%
- 100%
- Don’t know

10. **Across all age groups, how much greater is the risk of premature coronary heart disease (CHD) in untreated familial hypercholesterolemia patients compared to the general population?** ***Please answer to the best of your knowledge.***

- 2 times greater
- 5 times greater
- 10 times greater
- 20 times greater
- Don’t know

11. **When you are assessing a patient’s family history, at what age for males and females do you consider heart disease to be premature?**

- Premature heart disease in Males: ____ # Years of age or younger
- Premature heart disease in Females: ____ # Years of age or younger
- Don’t know

12. **In patients with documented premature coronary artery disease which one of the following do you routinely carry out?**

- Examine for arcus cornealis
- Examine for tendon xanthomata
- Take a detailed family history of coronary artery disease
- Screen close relatives for hypercholesterolemia
- All off the above
- None of the above

13. **An accurate diagnosis of FH can only be made via genetic testing.** ***Is this statement true or false?***

- True
- False
- Don’t know

14. **Which, if any, of the following have been recognized to further increase the cardiovascular risk of someone with familial hypercholesterolemia?** ***Please select all that apply.***

- Elevated Lipoprotein(a)
- Smoking
- Elevated C-reactive protein
- Type 2 Diabetes
- None of the above
- Don't know

15. **In your view, which healthcare providers would be <u>most effective</u> at early detection of familial hypercholesterolemia and screening first-degree relatives?** ***Please select up to two.***

- Lipid specialists
- PCPs
- Cardiologists
- Pediatricians
- OB/GYNs
- Endocrinologists
- Other (please specify)

16. **Have you ever diagnosed patient with FH?**

- Yes
- No

17. **Have you ever followed patient with FH?**

- Yes
- No
- Don't remember

18. **Which <u>one</u> of the following choices could help you in the detection of FH in your practice?**

- Laboratory comment on lipid profile alerting a possible FH
- Alert by the clinical software system in your practice
- Direct telephone call from the laboratory
- All of the above
- None of the Above

- Other (please specify)
- Don't know

19. **If you have patients with FH under your care do you routinely screen their close relatives for this condition with lipid profile?**

- Yes, patient's children only
- Yes, patient's children and other close relatives
- No
- Not applicable

20. **Are you aware of these Clinical diagnostic algorithms to diagnose patients with FH?**

The Simon Broome criteria	Yes	No
The Dutch Lipid Clinic Network DLCN criteria	Yes	No
The US MedPed Program	Yes	No

21. **At what age would you test young individual for hypercholesterolemia in a family with premature coronary heart disease?**

- 0-6 years
- 7-12 years
- 13-18 years
- None of the above
- Don't know

22. **Are you aware of the cascade screening for patient with familial hypercholesterolemia?**

- Yes
- No

23. **Are you aware of any specialist clinical services for lipid disorders to whom you can refer patients?**

- Yes
- No

24. **An LDL target for adults with FH is:**

- < 1.8 mmol/L (< 70 mg/dL)
- < 2.5 mmol/L (< 100 mg/dL)
- < 3.3 mmol/L (< 130 mg/dL)
- Don’t know

25. **An LDL target for FH adults with known CHD or diabetes is:**

- < 1.8 mmol/L (< 70 mg/dL)
- < 2.5 mmol/L (< 100 mg/dL)
- < 3.3 mmol/L (< 130 mg/dL)
- Don’t know

26. **Patients with FH should receive which one of the following medication as first-line treatment? Please select one option only.**

- Exchange resins / bile acid sequestrates
- Ezetimibe
- Statins
- Fibrates
- Nicotinic acid
- Don’t know

27. **Which drug combinations do you use to treat sever hypercholesterolemia?** ***Please select all that apply***

- Statin + Exchange resins / bile acid sequestrates
- Statin + Nicotinic acid
- Statin + Ezetimibe
- Statin + Ezetimibe+ Nicotinic acid
- Statin + Ezetimibe + Exchange resins / bile acid sequestrates
- Other (please specify)
- None of the above

28. **Are you aware to these medications for FH patients beside statins?**

○ PCSK9 inhibitors	○ Yes	○ No
○ Lomitapide (MTP) inhibitor.	○ Yes	○ No
○ Mipomersen (an antisense oligonucleotide inhibitor)	○ Yes	○ No
